# ICU management based on PiCCO parameters reduces duration of mechanical ventilation and ICU length of stay in patients with severe thoracic trauma and acute respiratory distress syndrome

**DOI:** 10.1186/s13613-016-0217-6

**Published:** 2016-11-21

**Authors:** Zhong Yuanbo, Wang Jin, Shi Fei, Long Liangong, Liu Xunfa, Xu Shihai, Shan Aijun

**Affiliations:** Emergency Center, Shenzhen People’s Hospital, Shenzhen, 518020 China

**Keywords:** Thoracic trauma, Acute respiratory distress syndrome, PiCCO, PaO_2_/FiO_2_, ICU stay

## Abstract

**Background:**

This study aimed to assess whether a management algorithm using data obtained with a PiCCO system can improve clinical outcomes in critically ill patients with acute respiratory distress syndrome (ARDS).

**Results:**

The PaO_2_/FiO_2_ ratio increased over time in both groups, with a sharper increase in the PiCCO group. There was no difference in 28-day mortality (3.2 vs. 3.6%, *P* = 0.841). Days on mechanical ventilation (3 vs. 5 days, *P* = 0.002) and ICU length of stay (6 vs. 11 days, *P* = 0.004) were significantly lower in the PiCCO group than in the CVP group. Treatment costs were lower in the PiCCO group than in the CVP group. Multivariate logistic regression model showed that the monitoring method (PiCCO vs. CVP) was independently associated with the length of ICU stay [odds ratio (OR) 3.16, 95% confidence interval (95% CI) 1.55–6.63, *P* = 0.001], as well as shock (OR 3.41, 95% CI 1.74–6.44, *P* = 0.002), shock and ARDS (OR 3.46, 95% CI 1.79–6.87, *P* = 0.002), and APACHE II score (OR 1.17, 95% CI 1.02–1.86, *P* = 0.014).

**Conclusions:**

This study investigated the usefulness of the PiCCO system in improving outcomes for patient with severe thoracic trauma and ARDS and provided new evidence for fluid management in critical care settings.

**Electronic supplementary material:**

The online version of this article (doi:10.1186/s13613-016-0217-6) contains supplementary material, which is available to authorized users.

## Background

Thoracic trauma is directly responsible for 25% of all trauma-related deaths and plays a major role in 25% of the remaining trauma deaths [1]. Among patients with severe thoracic trauma and acute respiratory distress syndrome (ARDS), which is usually accompanied by hypotensive shock, mortality would significantly increase if the patients receive massive blood or fluid transfusion [2]. Therefore, optimizing the management of fluid status is still a challenge in critical care. Indeed, severe pulmonary edema may result from fluid overload, which will lead to increased mortality [3, 4]. On the other hand, inadequate fluid volume will result in insufficient oxygen delivery due to low perfusion pressure, compromising patient prognosis. Therefore, it is important to monitor the fluid status of patients with thoracic trauma and ARDS.

Despite having been used for over 50 years, the usefulness of pulmonary artery catheters is disappointing [5]. The Pulse index Contour Continuous Cardiac Output (PiCCO) system from Pulsion Medical Systems (Feldkirchen, Germany) is based on transpulmonary thermodilution (TPTD) and continuous pulse contour analysis approaches. PiCCO is a minimally invasive technique and allows the monitoring of beat-by-beat cardiac output. In addition, volume status and pulmonary edema can be monitored, as well as the hemodynamic status [6]. The PiCCO system also allows for extravascular lung water (EVLW) monitoring [7]. Patients with acute severe thoracic trauma often have increased pulmonary EVLW. In addition, studies have demonstrated that ARDS is associated with elevated EVLW [8] and elevated EVLW is associated with an increased mortality rate [9, 10].

Optimizing the EVLW index (EVLWI) could be beneficial to patients with ARDS and severe thoracic trauma, but only one study investigated the outcomes of patients managed with PiCCO [11], while the other studies used intermediate parameters (fluid responsiveness, oxygenation, and pulmonary edema) only [12, 13]. Therefore, the present study aimed to examine the usefulness of a management algorithm based on the PiCCO system to improve the outcomes of patients with ARDS and severe thoracic trauma.

## Methods

### Study design

This study was performed prospectively and in consecutive 264 patients with severe thoracic trauma and acute respiratory distress syndrome (ARDS). All patients were admitted to the emergency intensive care unit (EICU) of Shenzhen People’s Hospital, China, between March 2010 and April 2014. Thus, study was approved by the Ethics Committee of the Shenzhen People’s Hospital. Written informed consent was obtained from the patients or their legal guardians/representatives.

### Patients

Inclusion criteria were: (1) adult patients (≥18 years old); (2) thoracic trauma; and (3) met the clinical criteria of ARDS within 24 h after admission to the EICU. ARDS was defined according to the Berlin definition [14]: (1) onset within one week of a known clinical insult or new/worsening respiratory symptoms; (2) bilateral opacities on chest imaging that could not be fully explained; and (3) the respiratory failure event could not fully be explained by cardiac failure or fluid overload. Exclusion criteria were: (1) <18 years old or >60 years old; (2) traumatic brain or spinal injury, cardiac trauma, intrathoracic major arterial or venous injury, or abdominal visceral injury; (3) was moribund or informed consent could not be obtained; (4) any contraindications to catheter insertion; or (5) vascular conditions leading to inaccuracies of PiCCO measurements (e.g., intracardiac shunts, significant tricuspid regurgitation, or cooling/rewarming) [15–17].

Different treatments were administrated when the patients arrived at the EICU, according to the type of thoracic trauma. Patients with flail chest received chest external fixation. Patients with hemothorax or pneumothorax received closed drainage of the pleural cavity. For patients with massive hemothorax, thoracotomy hemostasis could be performed. During operation, topical or unilateral pneumonectomy was carried out if the lung tissue was completely destroyed by the trauma. Thereafter, patients began to receive their subsequent treatment in EICU or floor ward. Blood gas was routinely tested every 12 h, and chest X-ray or CT was checked every day until 5 days after injury to evaluate whether ARDS occurred. As soon as ARDS was diagnosed, the patient was enrolled in this study, sent to the EICU, and managed with mechanical ventilation.

All patients were randomized to the PiCCO or CVP group using a randomization sequence generated with Stata 12.0 (StataCorp, College Station, TX, USA). Randomization was stratified according to the presence of shock and using a 1:1 ratio.

All electrocardiogram (ECG) measurements in the study were taken using a single ECG monitor (Philips IntelliVue Patient Monitor with a PiCCO module). In the PiCCO group, cardiac output and lung water were measured every 8 h. Investigators who collected the baseline characteristics and follow-up results were blinded to grouping.

### Interventions

In the PiCCO group, the PiCCO system was used within 2 h of enrollment. The aim of fluid management was to optimize the effective circulatory volume. If needed, hydroxyethyl starch 130/0.4 (Voluven^®^) and vasoactive agents were used to achieve a mean arterial blood pressure (MAP) of ≥60 mmHg. Diuretics were administrated to achieve a negative fluid balance, and PEEP would be increased when the volume status (ITBVI > 850 ml/m^2^) was optimized but with an EVLWI of ≥10 ml/kg. If there were a suspicion that circulatory failure was the result of cardiac dysfunction (CI less than 2.5 l/m^2^/min), dobutamine was started at 3.0 mg/kg/min. The use of the PiCCO system was discontinued after 48 h if the patient were clinically stable. Stability was determined by the attending physicians. Otherwise, the system was used for a maximum of 10 days.

For the patients in the CVP group, a central venous catheter was used, as per routine protocols. If the CVP was <8 mmHg, a 500-ml bolus of hydroxyethyl starch 130/0.4 (Voluven^®^) was infused over 20–30 min in order to achieve a CVP of 7–12 mmHg. The bolus was repeated if necessary. If the CVP exceeded 12 mmHg, the attending physician was allowed to use furosemide, at his discretion. If MAP was <60 mmHg, norepinephrine was infused at 0.05 μg/kg/min; the infusion could be increased by 0.05 μg/kg/min, at the discretion of the attending physician.

Therefore, the fluid management strategy was similar in the two groups. The main difference was the monitoring method. In the presence of a suspicion of catheter-related bloodstream infection (CRBSI), the central venous catheter was removed and analyzed to determine the causative agent, and a new catheter was indwelled.

The treatment algorithm was a circle that could be repeated if necessary, according to the condition of the patient. Without shock, volume expansion was not performed. The timing for the measurement of the hemodynamic parameters was at the discretion of the attending physician.

### Outcome measures

The PaO_2_/FiO_2_ ratio was calculated according to blood gas analysis. Mechanical ventilation was terminated if: (1) the patient were cooperative; (2) the patient were hemodynamically stable; (3) the patient had adequate and strong cough reflex; (4) the patient had positive end-expiratory pressure <5 cmH_2_O; (5) the patient had pressure support <10 cmH_2_O; and (6) the patient had a successful spontaneous breathing trial.

The ICU length of stay was defined from the day of EICU admission to the day of ICU discharge. If there were no longer any need for vital organ support, the patient was considered ready for discharge.

EICU cost for monitoring and treatment was determined according to the expenses from EICU admission to leaving ICU. Operation cost for thoracic trauma was excluded.

The 28-day mortality was defined as death from any cause before day 28. Adverse events were monitored, including hematoma, pneumothorax, arterial emboli, catheter-related bloodstream infection, hemorrhage, pseudoaneurysm, and arrhythmia.

### Statistical analysis

Normality of continuous variables was determined according to the graphical distribution of the values. Normally distributed continuous variables were analyzed using the one-sample *t* test for intergroup comparisons and using repeated measure ANOVA for intragroup analyses. Non-normally distributed continuous variables were analyzed using the Mann–Whitney *U* test. Categorical data were presented as frequencies and analyzed using the Pearson’s Chi-square test. Multivariate logistic regression was used to adjust for confounding variables. Variables that were statistically different between the PiCCO and control groups in univariate analyses (*P* < 0.05) were entered into a multivariate model. The efficacy of treatment based on PiCCO monitoring was investigated in subgroups of ARDS and/or shock. Stata 12.0 (StataCorp, College Station, TX, USA) was used for statistical analysis. Two-sided *P* values <0.05 were considered statistically significant.

## Results

### Characteristics of the patients

**Fig. 1:**
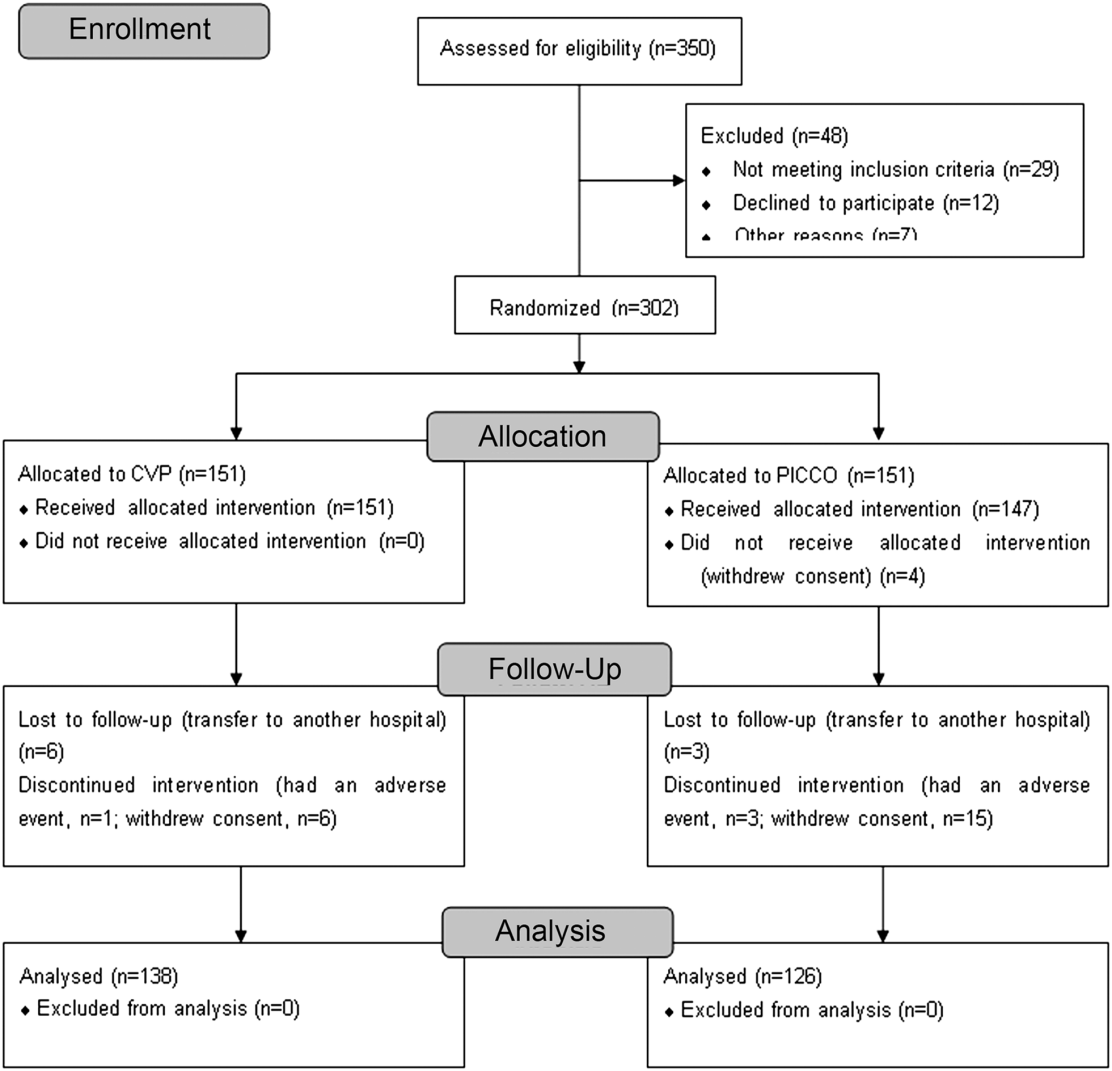
Patient flowchart

Figure 1 presents the patient flowchart. The baseline characteristics of the patients are presented in Table 1. The ARDS severity parameters are presented in Additional file 1: Table S1. There were 126 patients in the PiCCO group and 138 in the CVP group. The patients were more critically ill in the PiCCO group than in the CVP group (median APACHE II score, 27 vs. 23, *P* = 0.033; and median ISS score, 14 vs. 13, *P* = 0.038). The PiCCO group showed lower PaO_2_/FiO_2_ ratio (185 ± 58 vs. 209 ± 90 mmHg, *P* = 0.038). There were no differences between the two groups for gender, age, cause of injury, time between injury and EICU admission, shock, and hemoglobin levels (all *P* > 0.05).

**Table 1:** Characteristics of the patients at baseline

Characteristics	PiCCO group (*n* = 126)	CVP group (*n* = 138)	*P*
Male, *n* (%)	95 (75.4)	105 (76.1)	0.896
Age (years)	38.5 ± 7.6	37.9 ± 8.2	0.232
APACHE II, median (IQR)	27 (22–33)	23 (17–29)	0.033
ISS, median (IQR)	14 (12–16)	13 (12–15)	0.038
Type of injury cause, *n* (%)			0.578
Road traffic accident	66 (52.4)	71 (51.4)	
Falling injury	31 (24.6)	39 (28.3)	
Crush injury	20 (15.9)	19 (13.8)	
Explosive injury	9 (7.1)	9 (6.5)	
Time from acute onset to EICU admission, hours, median (IQR)	8 (2–31)	7 (2–24)	0.317
Shock, *n* (%)	90 (71.4)	94 (68.1)	0.559
ARDS severity^a^ (by PaO_2_/FiO_2_, *n* (%))			0.025
Mild (200–300)	7 (5.6)	8 (5.8)	
Moderate (100–200)	86 (68.3)	112 (81.2)	
Severe (<100)	33 (26.2)	18 (13.0)	
Hemoglobin (g/L)	85 (62–109)	87 (65–107)	0.883

*EICU* emergency intensive care unit, *IQR* interquartile range, *APACHE II* Acute Physiology and Chronic Health Evaluation II; *ISS* injury severity score

^a^According to the Berlin definition

### Outcomes

**Fig. 2:**
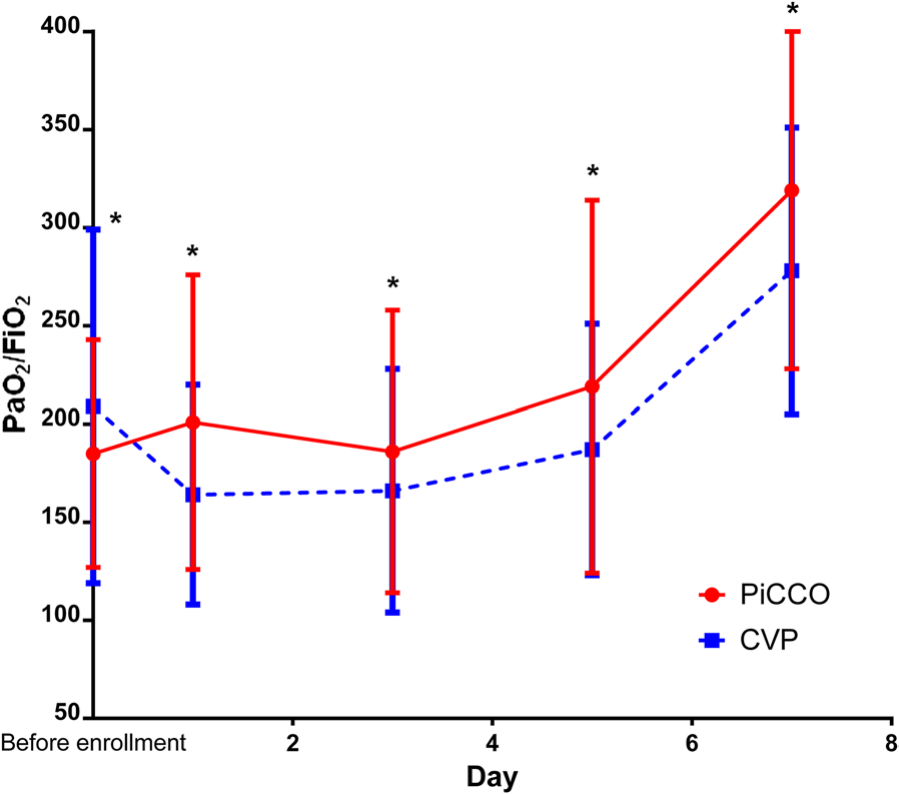
PaO_2_/FiO_2_ curve. PaO_2_/FiO_2_ values of PiCCO and CVP groups were collected from before enrollment to day 7. * PaO_2_/FiO_2_ ratio increased over time in both groups, with a sharper increase in the PiCCO group (Repeated measures ANOVA-analysis *P* = 0.002)

Fluid balance from day 1 to day 6 was similar between the two groups. On day 7, the amount of fluids received in the PiCCO group was significantly less than in the control group (median 188 vs. 644 ml, *P* = 0.028). Using repeated measures analysis, the test for a difference in PaO_2_/FiO_2_ ratio over time was statistically significant (*P* < 0.001) and the test for interaction between treatment and time was also significant (*P* = 0.002), indicating that the PaO_2_/FiO_2_ ratio increased over time in both groups, with a sharper increase in the PiCCO group (Fig. 2; Table 2), even though the PaO_2_/FiO_2_ ratio was lower in the CVP group on day 1.

**Table 2:** Comparison of outcomes between the PiCCO and CVP groups

Outcome variables	PiCCO group (*n* = 126)	CVP group (*n* = 138)	*P*
PaO_2_/FiO_2_			
Before enrollment	185 ± 58	209 ± 90	0.038*
1 day after enrollment	201 ± 75	164 ± 56	0.022*
3 day after enrollment	186 ± 72	166 ± 62	0.047*
5 day after enrollment	219 ± 95	187 ± 64	0.037*
7 day after enrollment	319 ± 91	278 ± 73	0.042*
Days on MV, days, median (IQR)	3 (1–6)	5 (2–9)	0.002
Length of stay in EICU, days, median (IQR)	6 (4–8)	11 (6–16)	0.004
EICU cost for monitoring and treatment (RMB: yuan)	8.23 ± 3.25	12.87 ± 4.61	<0.001
28-day mortality, *n* (%)	4 (3.2)	5 (3.6)	0.841

* Post hoc *p* value in repeated measure of ANOVA

*MV* mechanical ventilation, *EICU* emergency intensive care unit, *IQR* interquartile range

As shown in Table 2, there was no difference in 28-day mortality between the two groups (3.2 vs. 3.6%, *P* = 0.841). However, days on mechanical ventilation (median 3 vs. 5 days, *P* = 0.002) and ICU length of stay (median 6 vs. 11 days, *P* = 0.004) were significantly lower in the PiCCO group than in the CVP group. EICU monitoring and treatment also showed significantly lower cost in the PiCCO group than in the CVP group.

Some complications associated with the placement of the femoral artery catheter for the PiCCO system were encountered and included 11 cases of venous puncture (4.2%), 4 of hematoma (1.5%), 2 of guide wire kinking (0.8%), and one of catheter malfunction (0.4%).

### Multivariate analysis

The multivariate logistic regression model showed that the monitoring method (PiCCO vs. CVP) was independently associated with the length of ICU stay (odds ratio (OR) 3.16, 95% confidence interval (95% CI) 1.55–6.63, *P* = 0.001), as well as shock (OR 3.41, 95% CI 1.74–6.44, *P* = 0.002), shock and ARDS (OR 3.46, 95% CI 1.79–6.87, *P* = 0.002), and APACHE II score (OR 1.17, 95% CI 1.02–1.86, *P* = 0.014) (Table 3).

**Table 3:** Multivariate logistic regression model for the length of ICU stay

Length of ICU stay (bivariate: >7 days or ≤7 days)	Odds ratio	Lower limit of 95% CI	Upper limit of 95% CI	*P*
Group (CVP vs. PiCCO)	3.16	1.55	6.63	0.001
Gender (male as the reference)	1.16	0.59	1.74	0.863
Age (with 1 year increase)	1.11	0.98	1.21	0.536
Time from acute onset to ICU admission	0.98	0.94	1.13	0.237
PaO_2_/FiO_2_	0.79	0.46	1.37	0.283
Type of patient (ARDS as reference)				
Shock	3.41	1.74	6.44	0.002
Both	3.46	1.79	6.87	0.002
APACHE II	1.17	1.02	1.86	0.014
ISS	1.15	0.89	1.24	0.176

*APACHE II* Acute Physiology and Chronic Health Evaluation II, *ISS* injury severity score, *ER* emergency room, *OR* operating room

## Discussion

This study investigated the use of PiCCO-based or CVP-based fluid management for patients with severe thoracic trauma and ARDS. Results support the use of a PiCCO-based treatment algorithm. Indeed, the use of PiCCO significantly decreased the duration of mechanical ventilation and ICU length of stay without any major side effects. However, PiCCO-based fluid management did not improve mortality rate compared to CVP-based fluid management.

It is widely accepted and practiced in routine clinical practice that negative fluid balance benefits patients with ARDS [18]. Highly efficient diuretics have to be given if auscultation or chest X-ray suggests pulmonary edema and that ARDS is suspected. In the present study, the patients in the CVP group may actually experience similar levels of negative fluid balance despite unawareness of the exact amount of EVLW by the attending physician. Moreover, a substantial proportion of patients (>70%) had shock requiring massive blood or fluid transfusion on ICU admission for which the study protocol dictated positive fluid balance.

To the best of our knowledge, there are few studies exploring the effectiveness of treatment based on PiCCO-derived physiological values on outcomes of patients with severe thoracic trauma and ARDS. Goepfert et al. [19] compared the effect of PiCCO-based treatment in cardiac surgery patients to historical controls and observed that fluid management based on PiCCO shortened the length of stay in ICU, supporting the present study. Lenkin et al. [20] compared the outcomes of goal-directed therapy guided using PAC or PiCCO and observed that PiCCO-based treatment increased the volume of fluid therapy, improved hemodynamics and oxygen delivery index, and reduced the duration of mechanical ventilation after complex valve surgery compared with PAC-guided management. Furthermore, several studies conducted among sepsis/shock patients also showed the potential usefulness of EVLW-directed fluid therapy according to improvements of the duration of mechanical ventilation, length of stay in ICU, and mortality [7, 21–23]. However, two recent studies carried out in critically ill patients with sepsis and/or shock reported that PiCCO-based fluid management failed to improve outcomes [3, 24]. Surgical patients (including cardiac surgery and thoracic trauma patients) usually have better pulmonary and circulatory functions compared with patients with septic shock and/or ARDS, which could partly explain these discrepancies. Indeed, trials with positive conclusions were conducted almost 8–10 years ago when the beneficial effect of a restrictive strategy had not yet been established. Currently, the beneficial effect of negative fluid balance is pretty well known and high doses of diuretics are given at a certain EVLWI threshold, even if no PiCCO system was used. In addition, studies with negative conclusions enrolled more severely and critically ill patients. As we know, for this type of patients, fatal outcomes are difficult to reverse even though a PiCCO-based treatment algorithm is administrated. In the present study, the mortality rate was low, which could be due to the patients being young and without severe trauma. This could also explain the lack of a statistically significant difference between the two groups.

In the present study, the multivariate analysis revealed that the monitoring method (PiCCO vs. CVP) was independently associated with the length of ICU stay, as well as shock, shock and ARDS, and APACHE II score. These results suggest that more critically ill patients will stay longer in the ICU compared with patients less critically ill and that the use of PiCCO could help shortening the ICU stay in all patients, independently of the illness severity. An ongoing clinical trial will help confirming these results [25].

Several limitations have to be acknowledged. First, there was a difference in the final number of patients between the two groups because more patients had to be excluded from the PiCCO group. In addition, there was an imbalance in the severity score between the two groups. Patients in the PiCCO group were more severely ill than in the CVP group, which would influence the outcomes. Despite this, positive conclusions could still be observed. Second, the treatment approach based on hemodynamic monitoring was largely relying on clinical experience and it will have to be confirmed by additional studies. Third, only patients with thoracic trauma were included and the impact of a PiCCO-based fluid approach on more severe trauma such as thoracic trauma accompanied by brain injury or abdominal visceral injury is largely unknown. Fourth, specific treatments were triggered by specific values of hemodynamic variables (e.g., ITBVI less than 850 was used to trigger fluid bolus) and it must be highlighted that the normal ranges of physiological values from the PiCCO system are not fixed but vary among subjects [26]; the algorithm had to be modified to accommodate the clinical condition of each patient. In real-world settings, we suggest that the clinical condition and clinicians’ judgment should be considered rather than simply relying on PiCCO readings. Fifth, before admission, almost all patients had undergone operation and sedation. Therefore, it was often impossible to determine the real consciousness state of the patients. Therefore, we could only compute the APACHE II values according to what could be directly observed. Moreover, most patients (>90%) were directly sent to the operation room to receive emergency operation to control bleeding (damage control operation) and then were sent to the ICU. These patients who received emergency operation received 5 additional scores for APACHE II computation. Therefore, APACHE II scores could be overestimated. Lastly, the mortality rate was lower than expected, which may compromise the generalizability of the results. Additional studies are required to assess the usefulness of the PiCCO system in the management of fluids in trauma patients.

## Conclusions

In conclusion, this study verified that the PiCCO system is able to improve outcomes for patient with ARDS and severe thoracic trauma. The results provide new evidence for fluid management in these patients.

## Additional file

**Additional file 1: Table S1.** Mechanical ventilation parameters during the first 7 days.

## Abbreviations

ARDS: acute respiratory distress syndrome
OR: odds ratio
TPTD: transpulmonary thermodilution technique
EVLW: extravascular lung water
EVLWI: EVLW index
ICU: intensive care unit
EICU: emergency intensive care unit
ECG: electrocardiogram
CRBSI: catheter-related bloodstream infection
