# Supplementary material for: ICU management based on PiCCO parameters reduces duration of mechanical ventilation and ICU length of stay in patients with severe thoracic trauma and acute respiratory distress syndrome
Source: Ann Intensive Care. 2016 Nov 21;6:113. doi: 10.1186/s13613-016-0217-6 (PMC5118373; doi:10.1186/s13613-016-0217-6)
Supplement: Supplementary file 1 — Additional file 1: Table S1. Mechanical ventilation parameters during the first 7 days. [file 13613_2016_217_MOESM1_ESM.docx]

**Supplemental Table S1.** Mechanical ventilation parameters during the first 7 days

| Parameters | Baseline | |  | | Day 1 | |  | | Day 3 | |  | Day 5 | |  | Day 7 | |
| --- | --- | --- | --- | --- | --- | --- | --- | --- | --- | --- | --- | --- | --- | --- | --- | --- |
| Median (IQR) | PICCO | CVP |  | | PICCO | CVP |  | | PICCO | CVP |  | PICCO | CVP |  | PICCO | CVP |
| Vt (ml/kg) | 8.6 (7.8-9.2) | 8.9 (7.9-9.3) | | 7.6 (7.4-7.7) | | 7.3 (6.4-8.2) | | 6.8 (6.4-7.0) | | 6.3 (5.2-7.4) | | 7.3 (7.1-7.6) | 6.8 (5.8-7.1) | | 8.8 (8.2-9.2) | 7.9 (6.7-9.3) |
| RR | 21 (19-22) | 22 (19-24) | | 24 (22-26) | | 27 (23-29) | | 28 (26-31) | | 32 (26-38) | | 25 (22-29) | 29 (21-34) | | 20 (16-23) | 25 (17-29) |
| Pplat (cmH2O) | 32 (30-33) | 32 (30-33) | | 29 (27-32) | | 32 (30-33) | | 28 (26-34) | | 33 (28-36) | | 29 (28-31) | 31 (28-35) | | 30 (28-32) | 29 (24-32) |
| PEEP (cmH2O) | 5 (4-8) | 6 (4-8) | | 6 (4-9) | | 10 (5-13) | | 9 (7-11) | | 12 (7-14) | | 4 (3-8) | 8 (4-12) | | 1 (0-3) | 6 (3-8) |
| Driving pressure (cmH2O) | 27 (26-28) | 26 (25-28) | | 23 (21-26) | | 22 (19-25) | | 18 (16-21) | | 22 (17-26) | | 25 (22-27) | 20 (16-25) | | 29 (18-32) | 23 (21-25) |

Vt: tidal volume; PEEP: positive end expiratory pressure; Pplat: platform pressure; RR: respiratory rate
